# Mechanisms and risk factors of metabolic syndrome in children and adolescents

**DOI:** 10.1007/s12020-023-03642-x

**Published:** 2023-12-22

**Authors:** Valentina Codazzi, Giulio Frontino, Luca Galimberti, Andrea Giustina, Alessandra Petrelli

**Affiliations:** IRCCS Ospedale San Raffaele, Milan, 20132 Italy

**Keywords:** Metabolic syndrome, Children, Obesity, Inflammation, Gut microbiota, Hypertension

## Abstract

Metabolic syndrome (MetS) is a complex disorder characterized by abdominal obesity, elevated blood pressure, hyperlipidemia, and elevated fasting blood glucose levels. The diagnostic criteria for MetS in adults are well-established, but there is currently no consensus on the definition in children and adolescents. The etiology of MetS is believed to involve a complex interplay between genetic predisposition and environmental factors. While genetic predisposition explains only a small part of MetS pathogenesis, modifiable environmental risk factors play a significant role. Factors such as maternal weight during pregnancy, children’s lifestyle, sedentariness, high-fat diet, fructose and branched-chain amino acid consumption, vitamin D deficiency, and sleep disturbances contribute to the development of MetS. Early identification and treatment of MetS in children and adolescents is crucial to prevent the development of chronic diseases later in life. In this review we discuss the latest research on factors contributing to the pathogenesis of MetS in children, focusing on non-modifiable and modifiable risk factors, including genetics, dysbiosis and chronic low-grade inflammation.

## Introduction

Metabolic syndrome (MetS) is a complex disorder characterized by abdominal obesity, elevated blood pressure, hyperlipidemia and elevated fasting blood glucose levels [1]. Definitions of MetS in adults have been proposed by several organizations, leading to a consensus on the presence of 3 of the following 5 criteria for its diagnosis: (i) elevated waist circumference with cut-off based on the reference organization; (ii) blood pressure ≥130/85 mmHg or ongoing treatment for hypertension; (iii) fasting blood glucose ≥100 mg/dL or ongoing treatment for diabetes; (iv) triglycerides (TGs) ≥150 mg/dL; (v) high-density lipoprotein (HDL) cholesterol <40 mg/dL in males and <50 mg/dL in females or ongoing treatment for dyslipidemia. Currently, there is no consensus on the definition of MetS in children and adolescents and the diagnosis is based on a combination of clinical findings and laboratory tests.

Several definitions have been proposed for children, with most of them being adult definitions modified for pediatric patients (Table 1) [2–6]. Despite these efforts, a comprehensive definition that considers variations in fat distribution and insulin sensitivity related to age, gender, and ethnicity remains elusive. Tropeano et al. have comprehensively reviewed proposed definitions for MetS in pediatrics and asserted that the definition put forth by the International Diabetes Federation (IDF) is preferable, emphasizing its practical applicability in clinical settings [7]. The IDF has defined MetS for children aged 10 to 16 in a manner similar to its adult definition. The only differences are that for adolescents, ethnic-specific waist circumference percentiles are used, and there is a single cut-off level for high-density lipoproteins, instead of a gender-specific cut-off [8, 9]. For those aged 16 and above, the adult guidelines are applicable. However, based on these criteria, metabolic syndrome cannot be diagnosed in children under 10. Nonetheless, it is advisable to monitor their waist circumference if it exceeds the 90^th^ percentile. Moreover, in a large cohort of patients and using different MetS definitions, Koskinen et al. showed that childhood MetS and overweight are associated with an over 2.4-fold risk for adult MetS from the age of 5 onward [10]. They also found that a higher body mass index (BMI) during adolescence increases the risk of death from cardiovascular causes in adulthood [11]. Furthermore, an increased risk of silent coronary artery disease in adult patients with type 2 diabetes (T2D) has been linked to MetS, and the association with MetS was greater than that with its individual components. [12]. Due to the strong impact on life quality and the growing number of young individuals with MetS, estimated at around 25.8 million children and 35.5 million adolescents in 2020 [13], the purpose of this article is to review different factors involved in the pathogenesis of MetS with a focus on mechanisms described in children and adolescents.

**Table 1:** Comparison between definitions of Metabolic Syndrome in children

	Zimmet et al. (IDF Definition 10–16) [2]	de Ferranti et al. [3]	Cook et al. [4]	Viner et al. [5]
Defining criterion	≥3 criteria	≥3 criteria	Obesity and at least 2 of remaining 4 criteria	≥3 criteria
Obesity	WC ≥90^th^ percentile (age and sex specific, NHANES III)	WC >75th percentile	WC ≥90^th^ percentile or adult cutoff if lower	BMI ≥ 95^th^ percentile (age and sex specific)
Glucose intolerance	Fasting glucose ≥110 mg/dL (≥6.1 mmol/L)	BP ≥90^th^ percentile (age, sex, and height specific)	Fasting glucose ≥100 mg/dL (>5.6 mmol/L) or Known type 2 diabetes mellitus	Fasting hyperinsulinaemia or Impaired fasting glucose ( ≥ 6.1 mM/L) or impaired glucose tolerance: glucose at 120 min ≥7.8 mM/L
Dyslipidemia (triglycerides)	Triglycerides ≥110 mg/dL	Triglycerides ≥100 mg/dL	Triglycerides ≥150 mg/dL	Triglycerides ≥1.75 mM/L or HDL-C < 0.9 mM/L or total cholesterol ≥ 95^th^ centile
Dyslipidemia (HDL-C)	HDL-C ≤ 40 mg/dL (1.03 mmol/L; all ages and sexes, NCEP)	HDL-C ≤ 50 mg/dL (1.3 mmol/L)	HDL-C < 40 mg/dL (1.03 mmol/L)	
High blood pressure	BP ≥90^th^ percentile (age, sex, and height specific)	BP >90th percentile	Systolic BP ≥ 130 mm Hg or diastolic BP ≥ 85 mm Hg or treatment of previously diagnosed hypertension	Systolic BP ≥ 95^th^ percentile (age and sex specific)

*BMI* body mass index, *BP* blood pressure, *HDL-C* high-density lipoprotein cholesterol, *NHANES III* third National Health and Nutrition Examination Survey, *WC* waist circumference

## Epidemiology of MetS in childhood

MetS is becoming increasingly common in children and adolescents and the disparity in consensus makes it challenging for clinicians to compare studies that employ different diagnostic standards. Data obtained by de Ferranti et al., using an adapted children definition of Adult Treatment Panel from the Third National Health and Nutrition Examination Survey (NHANES III), identify a prevalence of 9.2% of children with MetS while nearly 64% of children present at least one metabolic alteration [3]. The ambiguity in using different definitions became evident in a recent comparative study of Reisinger C et al. where prevalence of pediatric MetS ranges between 2.1% using the IDF definition and 11.2% using Ferranti’s definition [14]. There is however a consensus on the most frequent metabolic alterations, where central obesity and dyslipidemia are the major determinants.

## Pathophysiology of MetS

Hypertrophic adipocytes, as consequence of visceral fat expansion during obesity, secrete inflammatory molecules which, coupled with the reduced insulin-mediated lipolysis suppression, results in increased release of circulating free fatty acids (FFAs). FFAs accumulate in ectopic sites leading to lipotoxicity in pancreatic β cells and inhibition of insulin signaling in liver and muscles [6, 15–36].

The mechanism undergoing IR in muscle is based on the competition of FFAs with glucose as energy substrate and the reduced expression of the glucose transporter 4 (GLUT4) [37]. IR in the liver is limited to glucose production, while insulin-induced lipolysis is preserved. This leads to an increased synthesis of triglycerides, which are secreted into the circulation as atherogenic VLDLs causing dyslipidemia and increased risk of cardiovascular complications [6, 16, 17, 35, 38, 39]. Increased glucose production from hepatocytes and concomitant decreased glucose uptake in skeletal muscle cells lead to hyperglycemia [15, 17, 33]. Moreover, the concomitant presence of proinflammatory signals activated in obesity, vasoconstriction induced by FFAs and decreased insulin-mediated vasodilation may also explain hypertension [17, 35]. The underlying mechanism has been proposed to be mediated by urotensin-II (U-II), a potent vasoconstrictor, whose serum levels are positively associated with hypertension, IR, inflammation and with the clinical outcome of T2D and cardiovascular disease [40–42].

## Non-modifiable risk factor of MetS: genetic predisposition

**Fig. 1:**
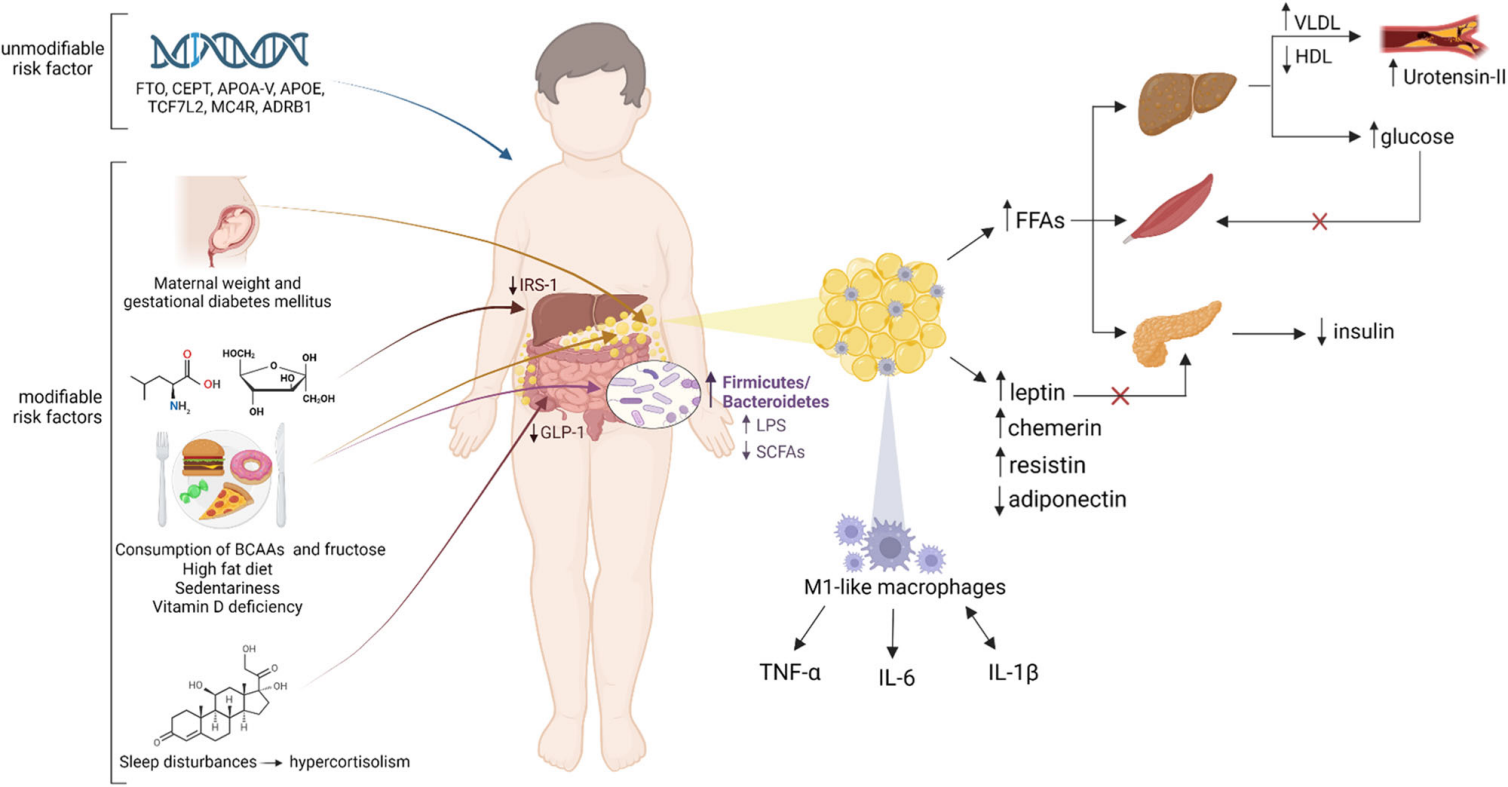
Pathogenesis of MetS in children and adolescents. Risk factors and mechanisms underlying the pathophysiology of MetS in pediatric individuals are depicted. Genetic predisposition and unhealthy lifestyle increase the risk for obesity and associated dysbiosis and inflammation which, in turn, lead to (1) increased production of proinflammatory cytokines by immune cells in the adipose tissue, (2) altered secretion of adipokines, (3) increased oxidative stress and vascular damage, (4) increased lipolysis and FFAs released from adipocytes, which lead to decreased responsiveness of insulin-sensitive tissues. As a result, insulin resistance, dyslipidemia and hypertension occur, increasing the risk of T2D and cardiovascular diseases. FTO fat mass and obesity-associated gene, CETP cholesteryl ester transfer protein gene, APOA-V apolipoprotein A-V gene, APOE Apolipoprotein E gene, TCF7L2 Transcription factor 7-like 2, MC4R melanocortin-4 receptor gene, ADRB1 beta-1 adrenergic receptor, BCAAs branched-chain amino acids, IRS-1 insulin receptor substrate 1, GLP-1 glucagon-like peptide-1, LPS lipopolysaccharides, SCFAs short-chain fatty acids, FFAs free fatty acids, VLDL very-low-density lipoprotein, HDL high-density lipoprotein, TNF-α: tumor necrosis factor alpha, IL-6 interleukin 6, IL-1β interleukin-1 beta

MetS is the result of a complex interplay between genetic predisposition and environmental factors. Figure 1 depicts risk factors and mechanisms underlying the pathophysiology of MetS in pediatric individuals. In Europe, the heritability estimates for MetS range from 10% to 30% depending on the diagnostic criteria used [43–45], meaning that genetic predisposition can explain only a small part of the pathogenesis of MetS. A large number of single-nucleotide polymorphisms (SNPs) have been described in relation to a single component of MetS; however, only few genes have been associated with MetS as a whole. In this direction, Povel et al. conducted a meta-analysis revealing the presence of the following SNPs in adult individuals with MetS: rs9939609 (*FTO*), rs7903146 (*TCF7L2*), *C56G* (*APOA5*), *T1131C* (*APOA5*), *C482T* (*APOC3*), *C455T* (*APOC3*) [46]. Similar data are lacking in children, where the most used approach is the investigation of a single SNP in one or more MetS traits.

The fat mass and obesity-associated gene (FTO) is located on chromosome 16 and plays a key role in weight control and energy balance [47]. Although the A/A phenotype in the risk allele rs9939609 (T/A) has been strongly associated with the occurrence of obesity [48], even in children [48–50], several lines of evidence suggest it plays a central role in the development of MetS [46, 51–54]. However, it’s not entirely clear whether the association with dyslipidemia is mediated by obesity [55].

The cholesteryl ester transfer protein (CETP) gene, also located on chromosome 16, may be equally involved in the pathogenesis of MetS [46]. CETP encodes for a protein involved in reverse cholesterol transfer; in fact, high levels of CETP result in lower circulation of HDL and consequent increases of low-density lipoproteins (LDL) and very low-density lipoproteins (VLDL). Adult MetS is inversely associated with the presence of the Taq-1B (rs708272) allele which, by reducing CETP expression, protects from dyslipidemia [46, 54, 56]. Heidari-Beni et al. confirmed the protective role of this polymorphism for MetS in children; however, they also demonstrated that the copresence of obesity restored, at least in part, CETP activity. Moreover, they identified another CETP polymorphism, A373P (rs5880), which increases the risk of dyslipidemia and related cardiovascular complications [56].

Apolipoproteins are responsible for lipid transport and some of them are encoded by genes whose polymorphisms have been associated with MetS [46, 57]. The SNPs T1131C (rs662799) on the APOA-V gene was correlated, both in adults and children, with high levels of triglycerides resulting from a lower ability to activate lipoprotein lipase [46, 58, 59]. Also, minor allele ε4 of the APOE gene induced the highest blood cholesterol and triglyceride levels if compared with major allele ε3 or another minor allele such as ε2 [46, 60–62].

The transcription factor 7-like 2 (TCF7L2) gene encodes for a protein that affects incretin-induced insulin secretion from pancreatic β cells [63] and confers the strongest genetic predisposition to the development of T2D. A study conducted on Caucasian and Asian adolescents associated the C allele of rs10749127 SNP with different features of MetS. A meta-analysis of five studies showed that the rs7903146 T allele also increased the risk for MetS in an adult population [46, 64, 65]. Although this allele is common among Europeans, studies in childhood and adolescence are still lacking in European countries [66].

The melanocortin-4 receptor (MC4R) gene is critical for energy balance; it regulates food intake as well as satiety in the hypothalamus and exerts its function at the peripheral level by preventing excessive fat deposition [47, 63]. The presence of the most common mutation rs17782313 (T/C) has been associated with several MetS traits (higher BMI and weight, higher triglycerides and lower HDL) as well as MetS itself in adults [46, 47, 67–70], but only with BMI in children [50, 69–72].

The Arg389Gly (rs1801253) SNP of the ADRB1 gene, which encodes for the β1 adrenergic receptor, was found to be associated with childhood obesity and blood pressure control [73–75]. A study in a French adult cohort showed that the simultaneous presence of PPARγ mutations 12Ala and 1431 C (rs385606) were associated with an increased risk of MetS that was not substantiated when analysed separately [76].

It has to be noted that studies aimed at investigating associations of genes with single MetS disorders do not take into account that most genetic loci have a pleiotropic effect on more than one MetS component and thus explain more than a single phenotypic trait; for example, a quantitative genetic analysis conducted on Hispanic children with metabolic disorders found a pleiotropic effect among genes encoding for systolic blood pressure, waist circumference and glucose, whereas a negative correlation was observed between HDL and waist circumference genes [77]. Moreover, Kraja AT et al. identified 25 genes whose SNPs are associated with at least two metabolic traits of MetS and at least one marker of inflammation [78]. These findings suggest the importance of considering the disease as a whole; however, the lack of defined diagnostic criteria for MetS is a severe limitation, as data are often not comparable between studies. In this scenario, assessing each SNP’s impact on MetS risk would help quantify the role of genetic predisposition. Another limitation in estimating the heritability of MetS is the difficulty in identifying rare or minor SNPs (defined by a minor allele frequency lower than 5% and 0.5%, respectively) that appear to be prevalent in children with obesity [70].

## Modifiable risk factors of MetS in childhood and adolescence

### Early in life

Environmental factors, including behaviour of the mother during pregnancy and children lifestyle, can contribute to the early development of MetS. Susceptibility for MetS already begins before birth, as high maternal weight during pregnancy and associated gestational diabetes mellitus increase the risk to develop obesity and type 2 diabetes in the offspring [79, 80].

### Obesity, insulin resistance and sedentariness

A sedentary lifestyle, obesity and insulin resistance (IR) trigger MetS. The World Health Organization indicates that IR is the common antecedent to all manifestations of MetS [15, 16, 32, 81, 82]; other studies suggest that obesity is the trigger of MetS [10, 83]. Considering that obesity and IR are closely related and most often occur together, it is correct to assume that both play an essential role in the pathogenesis of MetS and that neither factor is sufficient by itself to determine all the metabolic complications. The Bogalusa Heart Study revealed that both childhood obesity and IR can predict adult MetS development, but after adjustment for insulin and BMI respectively, only obesity maintained a significant association [84]. Two different obesity-associated metabolic conditions, namely metabolically healthy obese (MHO) and metabolic unhealthy obese (MUO) have been described in children as well as in adults [85]. An MHO phenotype during childhood is more likely to be retained during adulthood [86, 87]. Furthermore, the conversion of MHO children to MUO is determined by the loss of insulin sensitivity [88, 89], thus corroborating the hypothesis that MetS begins with obesity but requires IR to develop [90]. It is important to note that a physiological and transient IR occurs during pubertal development, and it could accelerate the onset of MetS in a pre-existing state of obesity-dependent IR [16, 35]. The causal link between obesity and IR lies in the elevated levels of proinflammatory adipokines, such as IL-6 and TNF-α, released by adipose tissue following fat accumulation, which worsens tissue responses to insulin, thus resulting in T2D, dyslipidemia and hypertension [29, 35, 91–94]. Sedentariness and high-fat diet take part in the development of obesity while elevated consumption of fructose and branched-chain amino acids contributes to a state of IR through the serine phosphorylation of the insulin receptor substrate-1 (IRS-1) and the resulting decrease in hepatic insulin sensitivity [95–97]. In children, the most common metabolic alterations are obesity and dyslipidemia with low HDL levels, whereas hypertension and glucose intolerance develop later in life and are typical of adult MetS [98, 99]. Obesity and dyslipidemia are consequences of poor dietary habits, whereas the age-specific decrease in HDL levels could be due to an androgen-sensitive increase in hepatic lipase activity and the consequent increase of HDL catabolism [100].

### Vitamin D deficiency, sleep disturbances and hypercortisolism

Vitamin D deficiency in youth has been associated with the presence of MetS [101]; emerging evidence suggests that adequate vitamin D levels may offer potential protection against the onset of metabolic complications. This includes fostering improved glycemic control, enhancing vascular function and regeneration, and reducing reactive oxygen species, thereby mitigating the risk of T2D and cardiovascular events [102, 103]. Despite these promising indications, a recent meta-analysis examining the impact of vitamin D supplementation in overweight and obese children revealed that elevated 25(OH)D levels did not translate into clinically significant outcomes [104]. As a result, the controversy surrounding the effectiveness of supplementation treatment persists.

Moreover, sleep disturbances, namely insufficient sleep, poor sleep quality and/or insomnia and obstructive sleep apnea, induce cortisol production by the adrenal cortex, which leads to a higher caloric intake and fat accumulation in children [32, 35, 105–107]. A higher obstructive sleep apnea severity [108], alongside hypercortisolism [109], is also associated with a lower glucagon-like peptide 1 (GLP-1) response to a glucose challenge. This is because GLP-1 production is under circadian rhythm control and can be altered in the presence of sleep disturbances [15, 17, 28, 110].

Hypercortisolism, a condition typically associated with Cushing’s Syndrome (CS), can also arise in other disorders, including those with suboptimal control of diabetes and severe obesity [111]. These patients, when exhibiting symptoms congruent with CS, may be alternatively diagnosed with physiological hypercortisolism or pseudo-CS. It is important to note that the clinical presentations of these cases of physiological hypercortisolism often lack the cutaneous (predisposition to bruising, skin thinning, and fragility) or muscular (proximal muscle atrophy and weakness) hallmarks of CS. During the diagnostic process for CS, it’s essential to methodically exclude these differential diagnoses or disorders [112].

### Systemic and tissue inflammation

MetS is accompanied by a chronic low-grade inflammation that is ascribable to obesity and could increase the risk of cardiovascular diseases later in life, as children appear to be more sensitive to oxidative stress than adults [11, 83, 113]. This is supported by the evidence that diet-induced weight loss exerts anti-inflammatory effects, resulting in improvements in metabolic parameters, lipid levels, and cytokine profiles [114]. A central role in the development of inflammation is associated to the activation of Toll-like receptors (TLRs), which triggers inflammatory signaling pathways and leads to the release of cytokines [15–17, 25, 28, 32]. Obesity in children exhibits similar inflammatory-mediated mechanisms as in adults, with similarly altered levels of cytokines and adipokines and increased expression of TLR2 and TLR4 [15, 25, 32, 115]. Here, we will focus on the description of inflammatory markers that have shown changes in children with single or multiple MetS traits.

Leptin is an adipokine highly produced by adipose tissue in obese children [116, 117] and, despite the ‘leptin resistance’ occurring in obesity, some of its effects are retained: specifically, leptin stimulates the production of IL-6 and TNF-α, contributing to the low-grade-inflammation, as well as activating the sympathetic nervous system leading to hypertension [15, 17, 28, 35, 116–119]. In physiological conditions, leptin stimulates the oxidation of FFAs and the uptake of glucose, thus preventing the accumulation of lipids in non-adipose tissues. When the abundance of FFAs is no longer compensated by leptin activity, they are shifted to the nonoxidative metabolic pathway and detrimental metabolites able to induce β cells death are produced [26, 33, 34]. Another adipokine strongly related to the pathogenesis of MetS is adiponectin, which is known to exert a variety of protective functions on metabolism and to induce an anti-inflammatory effect via inhibition of TLRs and secretion of anti-inflammatory cytokines [15–17, 28, 29, 32]. Therefore, it is not surprising that adiponectin levels are low in obese children [116, 117, 120]. Chemerin, a novel adipokine that regulates adipocyte development and metabolic functions, is strongly associated to BMI. Moreover, it has been proposed as an early biomarker in children for the risk of developing MetS complications [121–123]. The adipokine resistin enhances macrophage secretion of TNF-α [124]. Moreover, the development of peripheral IR has been proposed to be caused by its excessive production [125]. Resistin, whose name was chosen because of its relationship with IR, was expected to be the link between obesity and T2D [126, 127]. However, further investigations showed contradictory results in adults [128, 129], whereas an increased production of resistin was consistently described in obese children [116, 130]. It would be interesting to elucidate if resistin can be a distinctive tract of children with obesity or MetS.

Another adipokine with glucogenic properties, asprosin, is emerging as a potential mediator of obesity and MetS in children. Asprosin is a hormone protein derived from profibrillin, secreted by white adipose tissue during fasting, and plays a role in the hypothalamic control of food intake as well as hepatic glucose release [131]. In adults, its serum levels have been correlated with various MetS features, including obesity, hypertriglyceridemia, elevated cholesterol levels, T2D and IR. However, conflicting data still exist regarding its association with these factors in children [132–134].

Immune cells are known to exert metabolic functions. M1-macrophages release high levels of TNF-α in obesity which, in turn, provide an inflammatory stimulus that increases the production of IL-6, leptin and plasminogen activator inhibitor-1 (PAI-1) [28, 116, 135]. TNF-α induces serine phosphorylation of IRS-1, decreases the expression of GLUT4 in hepatocytes and adipocytes, and stimulates FFAs synthesis, leading to the development of IR [16, 28, 92, 136]. On the other hand, IL-6 in the liver stimulates the production of C-reactive protein (CRP), the main acute-phase inflammatory molecule associated with childhood obesity, as well as atherosclerosis and cardiac events [16, 17, 116, 117, 121, 137, 138]. Moreover, IL-1β, which is another cytokine mainly secreted by macrophages, acts by decreasing insulin action on adipocytes and promoting ectopic fat accumulation [139, 140].

In a condition of hyperglycemia, glucose accumulates in endothelial cells and, together with advanced glycation end-products (AGEs) and FFAs, increases oxidative stress and induces vascular damage [141]. Several studies have consistently identified heightened levels of oxidative stress markers in obese children, linking them to an elevated risk of metabolic, cardiovascular, and renal complications [142–144]. Among them, the ratio between AGEs and their soluble receptor form (sRAGE) has been proposed as an early indicator of oxidative homeostasis dysregulation [145]. Notably, sRAGE functions as a decoy by impeding the binding of AGEs to the Receptor for AGEs (RAGE) on the cell surface, thereby averting inflammation. In children with impaired metabolism, plasma sRAGE levels exhibit variability based on BMI and on the number of MetS components [146]. Correspondingly, the AGEs/sRAGEs ratio increases in overweight and obese children [147].

Furthermore, the risk of thrombotic events in childhood is enhanced by the TNF-α-mediated release of PAI-1 [16, 28, 116]. In this context, the role of vasodilatory nitric oxide (NO) is less clear. Its production is stimulated by insulin and when IR occurs nitric oxide synthase is less activated [141]. However, increased levels of NO are observed in obese children. This paradox can be explained by the fact that excess NO reacts with the reactive oxygen species (ROS) in radical reactions, thus providing more oxidative stress [144, 148].

New markers for metabolic disfunction are microRNAs (miRNAs). MiRNAs are short, noncoding single strain RNA molecules that regulate post-transcriptional gene expression by binding to complementary miRNA. A large number of miRNAs are described to be associated with single components of MetS [17, 149, 150], but only a few of them appear to be linked to the syndrome as a whole. Among them, miR-Let-7e, miR-93 and miR-24-3p circulating levels have been found to be increased in children with MetS [149–151].

Chronic inflammation ascribed to obesity plays an important role in triggering the mechanisms that lead to insulin resistance and cardiovascular events. This highlights the importance of prevention at an early age, when an appropriate lifestyle - with a balanced diet and adequate physical activity - has the potential to probtect from long-term complications.

### Alteration of the gut microbiota

The human intestinal microbiota is composed by a large number of microorganisms with the vast majority of bacteria belonging to the Firmicutes, Bacteroidetes, Actinobacteria, Proteobacteria, Fusobacteria and Verrucomicrobia phyla [152, 153]. Table 2 shows bacteria that have been described to be altered in the gut microbiota of pediatric individuals with obesity or MetS-associated disorders.

**Table 2:** Bacteria that have been described to be altered in the gut microbiota of pediatric individuals with obesity or MetS-associated disorders

Phylum	Class	Order	Family	Genus	Health condition	References
Firmicutes (or Bacillota)	Bacilli	Lactobacillales	Lactobacillaceae	Lactobacillus ↑	O vs NW	[159]
	Erysipelotrichia	Erysipelotrichales	Erysipelotrichaceae ↑		OMS vs O and OMS vs NW	[154]
			Turicibacteraceae ↓	Turicibacter ↓	O + IR vs O + IS	[158]
			Coprobacillaceae	Catenibacterium ↑	OMS vs NW and O	[154]
	Clostridia	Clostridiales (or Eubacteriales) ↑	Oscillospiraceae or ruminococcaceae ↓		O vs NW	[162, 163]
				Faecalibacterium ↓ Species: Faecalibacterium Prausnitzii ↓	O vs NW	[164]
					O vs NW	[154]
			Lachnospiraceae	Coprococcus ↑	OMS vs NW	[154]
				Anaerostipes ↓	O + IR vs O + IS	[142]
				Lachnospira ↑	O vs NW	[162, 163]
			Christensenellaceae ↓		O vs NW	[163]
			Eubacteriales family XIII	Eubacterium brachy ↓	O vs NW	[158]
			Peptococcaceae ↑		O + IR vs O + IS	[158]
	Negativicutes	Acidaminococcales	Acidaminococcaceae	Phascolarctobacterium ↓	O vs NW	[154]
		Veillonellales	Veillonellaceae	Dialister ↓	O + IR vs O + IS	[158]
Bacteroidota	Bacteroidia	Bacteroidales ↑	Prevotellaceae ↓		O vs NW	[162]
				Prevotella ↓	O vs NW	[164]
			Bacteroidaceae ↑	Bacteroides ↓	O vs NW	[164]
			Tannerellaceae	Parabacteroides ↓ species: Parabacteroides distasonis↓	OMS vs O	[154]
			Porphyromonadaceae	Porphyromonas↑	O vs NW and OMS	[154]
			Rikenellaceae	Alistipes ↓	O vs NW	[163]
Verrucomicrobia	Verrucomicrobiae	Verrucomicrobiales	Akkermansiaceae	Akkermansia Species: Akkermansia muciniphyla ↓	O vs NW	[161, 164]
Pseudomonadota	Gammaproteobacteria	Enterobacterales	Enterobacteriaceae ↑		O vs NW	[161]
		Pasteurellales	Pasteurellaaceae	Haemophilus ↓	O + IR vs O + IS	[158]
	Deltaproteobacteria	Desulfovibrionales	Desulfovibrionaceae	Desulfovibrio ↓	O vs NW	[164]
Actinobacteria	Coriobacteriia	Coriobacteriales ↓			O + IR vs O + IS	[158]
			Coriobacteriaceae	Collinsella Species: Collinsella aerofaciens ↑	OMS vs O and NW	[154]
		Eggerthellales	Eggerthellaceae	Adlercreutzia ↓	O + IR vs O + IS	[158]

*NW* normal weight, *O* obese, *OMS* obese with metabolic syndrome, *O* *+* *IR* obese with insulin resistance, *O* + *IS* obese with insulin sensitivity

Metabolic syndrome has been associated with a higher Firmicutes/Bacteroidetes ratio (F/B) [154–156]. In studies conducted by Gallardo‑Becerra et al. [154] and Haro et al. [155], in children and adults respectively, a higher abundance of Firmicutes and a lower abundance of Bacteroidetes were found in patients with MetS. These differences were statistically significant when obese patients with MetS were compared with normal-weight subjects; however, obese patients without MetS also showed an increase in the F/B ratio [154, 156]. Given that obesity plays a central role in the development of MetS, it’s not surprising that dysbiosis goes in the same direction. However, as both obesity and IR have been independently associated with an increased F/B ratio in children [157–159], the timing of changes in gut microbiota composition during the natural history of MetS is unclear.

Within the Firmicutes phylum, an increase in the class Bacilli is observed in MetS children [154]. In an adult cohort of patients with MetS this was attributed to the expansion of the Lactobacillus genus [160]. However, conflicting data regarding the Lactobacillus genus are reported in obese children [159, 161]. In addition, in the order Erysipelotrichales, an increase in the genus Catenibacterium was observed in children with MetS [154], while a decrease in the Turicibacter genus was described in children with IR [158].

The most significant difference within the Firmicutes phylum is found in the class Clostridia, where the increase of the overall abundance of the Clostridiales order concomitant with metabolic traits in children [154] is due to an imbalance between several genera. Moreover, the obesity phenotype is associated in children with a lower presence of the Oscillospiraceae family [157, 162, 163] and specifically with a decrease in the Faecalibacterium prausnitzii species [154, 164]. These data are in line with those described in adults with MetS, with obesity being the driving factor of this alteration [155, 160]. Faecalibacterium prausnitzii has an anti-inflammatory function, and its decrease may be the result of a protracted inflammatory process, as occurs in obesity [164].

Several studies have shown that the decline in the phylum of Bacteroidota is due to significant changes in the order of the Bacteroidales. However, while the reduction in Prevotella appears to begin with the onset of childhood obesity [162, 164] and then persists in adult MetS [155], other bacterial genera have shown different characteristics between these two groups. Specifically, obesity was associated with a decrease in Alistipes and an increase in Phorphyromonas [154, 163], while Parabacteroides distasonis represents a biomarker of MetS [154, 155, 160] as it negatively correlates with more than a single metabolic disorder, including waist circumference, glucose and triglycerides serum levels [160].

Bacteria contribute to the development of MetS through several mechanisms. First, the gut microbiota contributes to low-grade inflammation through infiltration of lipopolysaccharides, causing endotoxemia and TLRs activation [17, 25, 28, 107, 153, 161]. In addition, dysbiosis is characterized by a reduction in short-chain fatty acids (SCFAs)-producing bacteria [155, 165, 166]. SCFAs are metabolites obtained from microbial fermentation of indigestible carbohydrates that protect against the development of metabolic abnormalities; they stimulate the production of molecules such as GLP-1 and GLP-2 which have an anti-inflammatory activity and improve the function of the intestinal barrier [153, 165, 167]. Although the gut microbiota may change with age and is sensitive to environmental factors -such as social status and diet-, its composition clearly changes between MHO and MUO children [153, 157, 159, 168]. Moreover, bacteria associated with the production of SCFAs, namely Parabacteroides distasonis, Prevotella and Faecalibacterium prausnitzii, as well as the F/B ratio, in adults with MetS follow the same trend as in children [153, 157, 165]. Benefits resulting from the restoration of gut microbiome composition through the administration of probiotics or fecal microbiome transplantation corroborate the crucial importance of having a healthy gut [28, 165, 169].

## Conclusions

MetS in childhood and adolescence is a risk factor for cardiovascular diseases and death in adulthood. Currently, there is no consensus on the definition of MetS in children and adolescents, especially for children younger than 10 years. The rise of MetS in childhood is influenced by various factors, including increased obesity, dietary changes, and a sedentary lifestyle. However, the lack of standardized diagnostic criteria for children leads to underdiagnosis and undertreatment. While genetic predisposition has limited influence on the development of MetS, modifiable risk factors, such as maternal weight during pregnancy, children’s lifestyle, obesity, IR, sedentary behaviour, vitamin D deficiency, sleep disturbances, hypercortisolism, chronic inflammation, and alterations in gut microbiota play a crucial role in the development of MetS. Current interventions emphasize increased physical activity and a healthy diet, with positive effects on MetS components, even in children [170]. Notably, regulatory agencies like the U.S. Food and Drug Administration (FDA) and the European Medicines Agency (EMA) recently approved glucagon-like peptide-1 receptor agonists for chronic weight management in pediatric patients aged 12 years and older. Despite progress, the debate continues on extending adult-tested pharmacological advancements to children, raising questions about addressing the therapeutic gap.

Achieving consensus on diagnostic criteria, implementing early prevention strategies, and addressing environmental factors are essential in influencing the natural history of MetS in childhood and adolescence.
